# Exploring peripheral fluid biomarkers for early detection of Alzheimer's disease in Down syndrome: A literature review

**DOI:** 10.1016/j.heliyon.2024.e41445

**Published:** 2024-12-24

**Authors:** Charlotte Jacob, Marleen Tollenaere, Hanane Kachar, Marie-Claude Potier, Peter Paul De Deyn, Debby Van Dam

**Affiliations:** a Laboratory of Neurochemistry and Behaviour, Experimental Neurobiology Unit, University of Antwerp, Belgium; b Department of Neurology and Memory Clinic, Hospital Network Antwerp (ZNA) Middelheim and Hoge Beuken, Antwerp, Belgium; c Department of Neurology and Alzheimer Center, University of Groningen and University Medical Center Groningen, Groningen, the Netherlands; d Institut du Cerveau, Pitié-Salpêtrière Hospital, Centre National de la Recherche Scientifique, Institut National de la Santé et de la Recherche Médicale, Sorbonne Université, Paris, France

**Keywords:** Blood, Urine, Neurofilament light chain, Monoaminergic biomarkers, DYRK1A, Cerebral amyloid angiopathy

## Abstract

People with Down Syndrome (DS) are at high risk of developing Alzheimer's disease dementia (AD) and cerebral amyloid angiopathy, which is a critical factor contributing to dementia in sporadic AD. Predicting and monitoring the decline and onset of dementia is a diagnostic challenge and of essence in daily care and support for people with DS. In this literature scoping review, we first summarize the different blood-based biomarkers for AD in DS. Next, we describe urine-based biomarkers for AD in DS and finally, we explore various blood-based biomarkers in the general AD population. Apart from the classic amyloid beta and Tau biomarkers, we also discuss more out-of-the-box biomarkers such as neurofilament light chain, Dual-specificity tyrosine-regulated kinase 1A, and monoaminergic biomarkers. These potential biomarkers could be a valuable addition to the established panel of fluid biomarkers.

## Highlights

• Predicting decline and onset of dementia is challenging in people with Down Syndrome.
• We need tools for earlier and more sensitive Alzheimer's diagnosis in Down Syndrome.
• Peripheral biomarkers for dementia could be a valuable addition to the established CSF biomarkers.
• Studying biomarkers in Down Syndrome may enhance dementia understanding in the general population.

## 1 Introduction

**Fig. 1:**
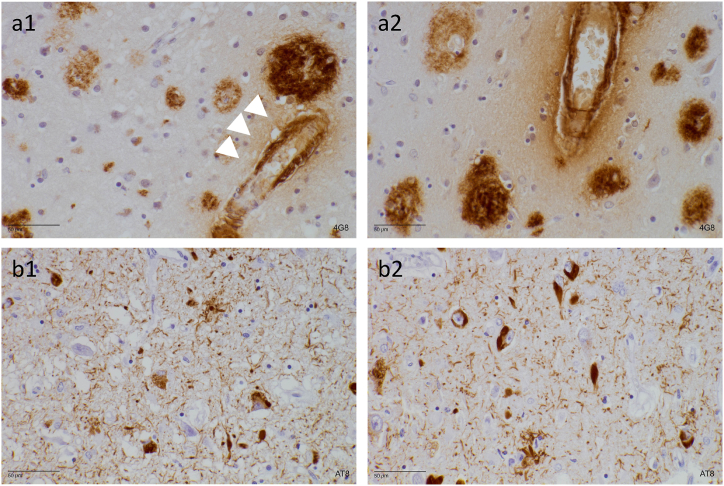
Amyloid and tau burden in Down Syndrome. Histological images showing Alzheimer's pathology in formalin-fixated and paraffin-embedded hippocampal brain tissue of a person with Down syndrome. The 4G8 antibody directed against amino acid residues 17–24 of amyloid-β was applied in panels a1 and a2, while the AT8 antibody directed against phosphorylated paired helical filament tau (serine 202 and threonine 205) was applied for panels b1 and b2. Hematoxylin was used as a nuclear counterstain. Panels a1 and a2: The extracellular senile plaques of aggregated amyloid-β appear as round brown structures. These plaques are relatively large and are positioned around and between the neurons, inhibiting cell communication. The white arrows indicate an accumulation of amyloid-β inside a blood vessel wall (cerebral amyloid angiopathy). Panels b1 and b2: The fiber-like brown structures represent neuropil threads. Neurofibrillary tangles of hyperphosphorylated tau protein are visible in the cell bodies of the hippocampus. Images used with permission from the Institute Born Bunge, University of Antwerp, Belgium.

Down syndrome (DS), caused by the (partial) triplication of human chromosome 21, is present in approximately 1 in 800 births, making it the most common genetic cause of intellectual disability in humans [1]. Individuals with DS furthermore face premature aging compared to the general population, and more specifically, also a significantly increased risk of developing early-onset Alzheimer's disease (AD) and cerebral amyloid angiopathy (CAA). By the age of 40, most people with DS will have developed significant AD-typical pathological alterations in the brain (see Fig. 1). The neuropathological changes are similar to those seen in persons with AD in the general population (i.e. Amyloid βeta (Aβ) plaques, neurofibrillary tangles of hyperphosphorylated tau protein, and neuroinflammation), but appear decades earlier [[2], [3], [4]]. Despite these changes, we observe that approximately 30 % of all people with DS in their 50s and 50 % of those in their 60s develop clinical AD (Alzheimer's [[4], [5], [6]]). The cumulative incidence of dementia in people with Down syndrome is more than 90 % by age 65 [7]. As such, it comes as no surprise that DS is currently considered the leading genetic risk factor for early-onset AD. The triplication of chromosome 21, and consequently the triplication of the amyloid precursor protein (APP) gene causes an overproduction of APP protein and its secretase product Aβ from birth onwards. This is probably the main cause of the DS population's strongly increased risk for AD and CAA. However, Ovchinnikov and colleagues (2018) have demonstrated that despite the deletion of the supernumerary copy of the *APP* gene in an isogenic DS human model (where they deleted the supernumerary copy of the *APP* gene in trisomic Down syndrome induced pluripotent stem cells or upregulated *APP* expression in euploid human pluripotent stem cells using CRISPRa) tau pathology still emerged. This research challenges the idea that 1.5 copies of *APP* is the sole reason for the increase of specific phosphorylated forms of tau in AD in DS individuals [8,9], although pure tauopathies do not seem to occur in DS individuals. Another important protein in both DS and AD is Dual-specificity tyrosine phosphorylation-regulated kinase 1A (DYRK1A). DYRK1A is known to hyperphosphorylate Tau, which increases the cellular aggregation of Tau, contributing to the formation of neurofibrillary tangles (NFTs) [10]. A substantial number of DS individuals will however never develop clinical AD throughout their life. Why one individual with DS clinically develops dementia while the other does not despite the presence of pathological AD brain hallmarks remains to be elucidated [11].

Predicting the onset and progression of dementia in DS is necessary for caregivers and relatives to understand the clinical change within patients and plan for adaptive daily caregiving. We need valuable tools that contribute to more sensitive and earlier diagnoses, such as an objective biomarker panel for AD in DS. The fact that cerebrospinal fluid (CSF) sampling via lumbar puncture is a relatively invasive procedure and rather burdensome, particularly for mentally fragile individuals like the DS population, underlies the growing interest in peripheral fluid biomarkers. In addition, studying biomarkers will improve our knowledge of the pathophysiology of AD in DS and may even aid the understanding of dementia mechanisms in the general population. In later stages, the identification of relevant biomarkers can contribute to target discovery for the development of novel efficacious drugs as preventive or curative treatment of AD [12]. This scoping review aims to summarize current knowledge on peripheral fluid biomarkers of AD in DS. After sections focusing on the diagnosis of AD in DS, and especially the limitations of the diagnostic approaches in the general population for application in people with DS, we present the current state of knowledge concerning blood- and urine-based biomarkers. The main focus lies on biomarkers for AD in DS, nevertheless, a brief statement regarding the current state of the discussed biomarker in the general AD population is included.

## 2 Diagnosis of Alzheimer's disease in Down syndrome

### 2.1 The challenging diagnosis of Alzheimer's disease in Down syndrome

Diagnosing AD or other neurodegenerative disorders causing cognitive decline is relatively complex, even in the general population. In current clinical practice, AD diagnosis in the general population is based on a multidisciplinary approach including medical history, mental status, and neuropsychological evaluation in combination with biomarker evidence for AD-typical pathology obtained via neuroimaging (e.g. MRI-based evidence of atrophy, PET measures indicative of neurodegeneration-related hypometabolism or Aβ deposition) or biofluid analysis (decreased CSF Aβ and increased T-tau or P-tau CSF levels) [13].

Diagnosing and monitoring the progression of clinical AD in DS presents even greater complexity considering the variable clinical onset and comorbidities that might be misinterpreted as dementia symptoms [14]. A variety of other disorders, like depression, thyroid dysfunction, hearing impairment, and pain, can cause apparent cognitive decline in DS and need to be excluded beforehand, to be able to diagnose AD [15].

Similar to AD in the general population, the clinical diagnosis of AD in DS is usually based on a multidisciplinary approach with clinical observations and caregiver reports concerning a patient's baseline functioning. The use of specific questionnaires (such as the Dementia Questionnaire for People with Learning Disabilities, DLD) and neuropsychological assessments are recommended [7,11,40]. Before the presentation of cognitive decline, people with DS mostly present with alterations in behavior and personality, defined as Behavioral and Psychological Symptoms of Dementia (BPSD). Recently, the first DS-specific BPSD (i.e. BPSD-DS) scale was developed, which may serve as a non-invasive monitoring tool to indicate DS subjects at risk of developing dementia at an early stage [[16], [17], [18]]. Objective biomarker profiles allowing for the prediction of conversion to AD are nevertheless still lacking [19,20].

The use of neuroimaging techniques or CSF biomarker analysis to support a diagnosis of AD in DS, however, is challenging, as these individuals often suffer from multiple phobias and generalized anxiety and these techniques are relatively invasive. Neuroimaging using Pittsburg compound B (i.e. amyloid load measured by PET) is moreover irrelevant to diagnose AD in DS because essentially every DS individual will be amyloid-positive depending on the individual's age [21].

Since CSF is in direct contact with the extracellular space of the brain, and also has a clearance function, CSF biomarkers are considered valid indicators of brain-specific activities and central nervous system pathology, rendering it the often-preferred source of AD biomarkers [22,23]. However, CSF is only obtainable through a lumbar puncture procedure, which may be regarded as invasive and complicated, and raise ethical issues, especially in the vulnerable DS population [24,25]. Although the classic CSF biomarkers are not easily applicable in DS, they have been investigated in a few studies [24,25]. Plasma or serum studies in DS are still in their infancy, but candidate biomarkers have already been identified [12,[26], [27], [28], [29]].

### 2.2 Biomarkers for Alzheimer's disease: moving towards valid blood biomarkers

Given the invasiveness, cost, and potential limited availability of neuroimaging and CSF biomarker analyses, other potential sources of AD fluid biomarkers have been contemplated. Biofluids like plasma, serum, and urine, are more routinely available and collection of these fluids is considered as a relatively non-invasive procedure [30,31]. As an additional advantage, these peripheral biofluids do not require a hospital setting and can be collected in the comfort of the patient's own home. On the other hand, the blood levels of brain-specific proteins reflecting AD mechanisms are often much lower than the levels in CSF, requiring the concurrent development of ultrasensitive analytical approaches, such as neuron-derived extracellular vesicles (in blood samples) and Single Molecule Array (Simoa®) [32,33].

Whereas the core AD CSF biomarkers have become an important element in the differential diagnostic procedure of dementia [23], medical research is now concentrating on novel candidate CSF biomarkers, and, in addition, on peripheral biomarkers. Several novel blood-based biomarkers, among which Aβ and tau protein [[34], [35], [36], [37]], glial fibrillary acidic protein (GFAP) [38], microRNAs [39,40], plasma proteins [41,42], as well as inflammatory cytokines [43,44] have been proposed, but large-scale longitudinal studies are needed for further verification and validation. A promising future blood biomarker is neurofilament light protein (NfL), an axonal neuron-specific protein increased in AD and Mild cognitive impairment (MCI) patients, but also in other neurodegenerative disorders such as frontotemporal dementia (FTD) [[45], [46], [47]]. Furthermore, several studies investigate biomarkers in noninvasive samples, such as urine, saliva, hair, and nails. Some promising peripheral biomarkers are found in urine, like isoprostane 8,12-iso-iPF2a-VI, total free amino acids, 8-hydroxy-2-deoxyguanosine, and glycine [48].

## 3 Peripheral fluid biomarkers for Alzheimer's disease in Down syndrome

PET and CSF biomarker analyses are advancing quickly [49]. They are, however, limited in their use as first-line diagnostic tools. Despite a vast amount of reported evidence from single-centre and multicentre trials and meta-analyses that support the use of neuroimaging biomarkers, their uptake in clinical practice and reimbursements from insurers vary substantially across the world, hence, hampering routine application. Blood-based biomarkers show therefore great promise [50], also in the DS population. Blood can also easily be retaken after time intervals, making longitudinal studies more accessible than with for example CSF testing [51]. A second advantage would be the cost of the sampling. PET scans are quite expensive and therefore less easily used. Finally, the use of blood-based biomarkers offers the potential for testing an elaborate range of exploratory and candidate biomarkers, reflecting the full spectrum of disease-triggering and driving mechanisms [52]. Urine is attractive for biomarker discovery as it is easily accessible and sampling can be performed in a non-invasive manner and performed repeatedly. Urinary biomarkers can help in providing first-line screening of diseases for a larger population, which can be confirmed through more sensitive CSF and blood-based biomarker analyses with high reliability. Due to the lack of any homeostasis mechanism, urine might reflect pathological changes, especially in the early stages of neurodegenerative diseases [53].

In the subsequent sections, biomarkers related to AD hallmarks and neurodegeneration (section 3.1), neuroinflammation (section 3.2), oxidative stress (section 3.3), monoaminergic and amino acid neurotransmitter changes (section 3.4), and epigenetic changes (section 3.5) are presented. These biomarkers have all been evaluated in DS populations.

### 3.1 Peripheral biomarkers linked to Alzheimer's pathology and neurodegeneration

Blood-based biomarkers linked to amyloid and tau-pathological hallmarks, as well as neurodegeneration, have been identified as cost-effective and scalable alternatives to imaging and CSF markers of AD.

#### 3.1.1 Amyloid-β protein

The extracellular deposits of the Aβ protein (Aβ_1-40_ and Aβ_1-42_), known as senile or neuritic plaques, are one of the two neuropathological hallmarks of AD [54]. The levels of both Aβ_1-40_ and Aβ_1-42_ can be measured by techniques such as an enzyme-linked immunosorbent assay (ELISA), Meso Scale Discovery Immunoassay (MSD) and Simoa®.

It is common knowledge within the field that Aβ concentrations in CSF are lower in AD patients than in healthy controls [55]. This has also been shown in blood [56].

As for now, only studies regarding Aβ in blood have been published for AD in DS. Several studies have investigated the applicability of blood Aβ as a biomarker for dementia in the DS population [[57], [58], [59]]. Compared to the general population, DS individuals showed higher plasma Aβ_1-40_ and Aβ_1-42_ levels (ELISA, MSD, or Simoa®). However, when investigating the changes in plasma Aβ concentrations in relation to cognitive tests and the status of dementia, studies report contradictory results [59,60]. It has to be noted however, that platelets produce 90 % of the peripheral Aβ. This could potentially interfere with blood-based assays [61].

#### 3.1.2 Tau protein

Tauopathy encompasses the degeneration of neurofibrils, leading to neuronal dysfunction and dementia.

According to the ATN criteria, total tau is recognized as a biomarker for neurodegeneration in cerebrospinal fluid (CSF). However, this connection has not been effectively observed in blood [62]. Plasma total tau levels show a poor correlation with CSF levels, likely due to the peripheral production of total tau, and they lack specificity for AD [63]. In the general population, plasma p-tau levels progressively rise along the continuum of sporadic AD, correlating with the severity of Aβ pathology and cognitive function [64].

**Fig. 2:**
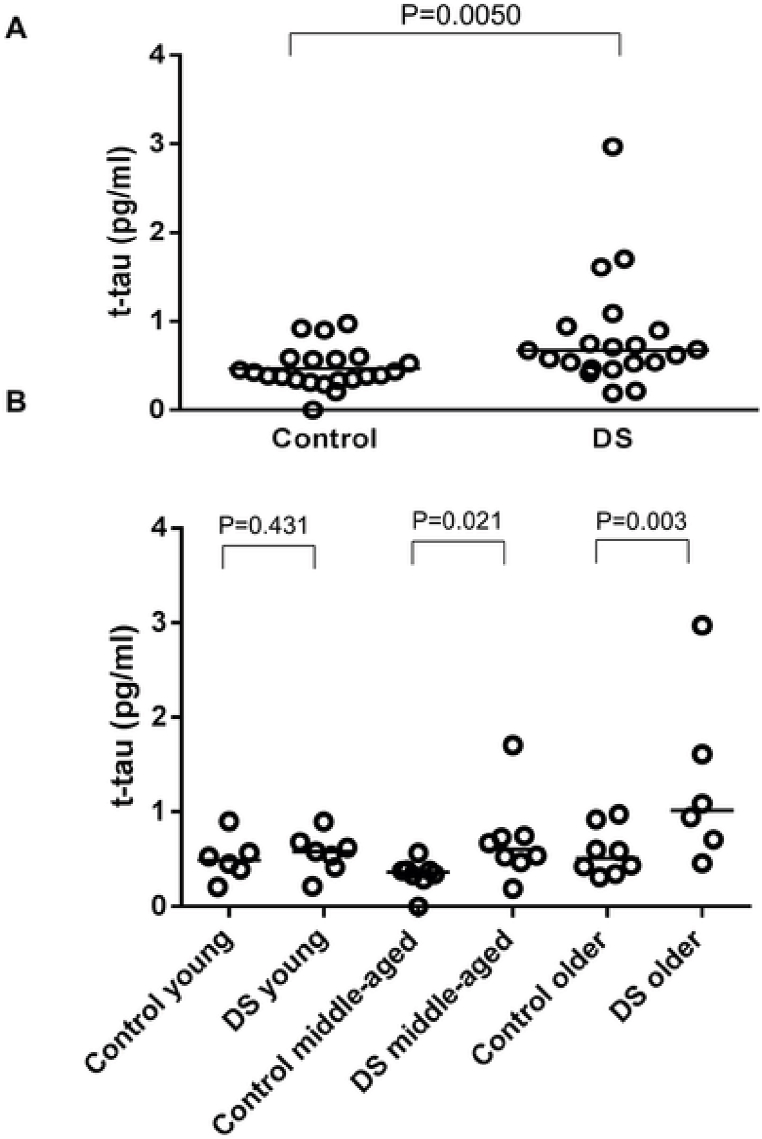
Tau burden in Down syndrome individuals versus healthy control individuals. (A) Scatter plot for total tau (t-tau) level in plasma in a group of non-DS (control group) (n = 22) and DS individuals (n = 21). Bars indicate median values. Levels of t-tau in the DS group were significantly higher than those of the control group (P = 0.0050). (B) Scatter plot for t-tau level in plasma in young (ages 14–25 years; n = 6 in the control group and 7 in the DS group), middle-aged (ages 26–42 years; n = 8 in the control group and 8 in the DS group), and older individuals (aged older than 43 years; n = 8 in the control group and 6 in the DS group). Bars indicate median values. Levels of t-tau in the DS group were significantly higher than those of the control group in the middle-aged and older group (P = 0.021 and P = 0.003, respectively). Abbreviation: DS = Down syndrome. Copied from Kasai et al. (2017) under the terms of the creative commons attribution license [68].

DS individuals have been reported to display higher plasma total tau (t-tau) levels compared to healthy control individuals, as tested with Simoa® (see Fig. 2) (Kasai et al., 2017). Furthermore, plasma tau levels correlated with cognitive scores and decreased after the onset of dementia. The lower levels of tau in DS with dementia may be explained by long-term neurodegeneration and a possible burn-out phenomenon of neurons (synaptic fatigue) [65]. In contrast, Janelidze and colleagues showed increased levels of plasma p-tau 217 in demented DS-individuals (whether it was a form of MCI or actual clinically diagnosed AD) when compared to cognitively stable DS individuals or non-DS siblings [66]. A similar pattern for p-tau 181 was demonstrated by Lleó et al. [67].

**Fig. 3:**
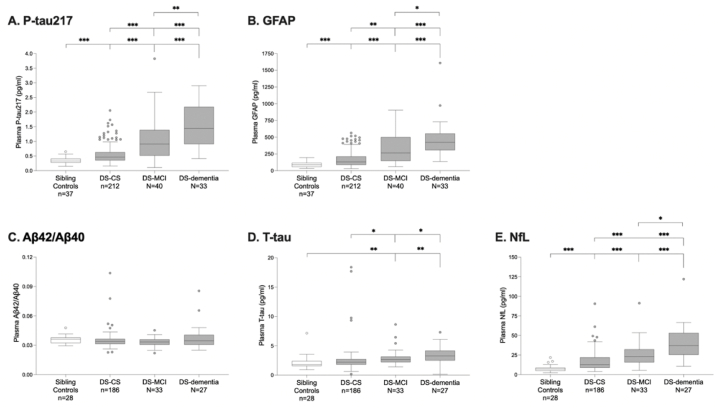
Plasma biomarker concentrations across diagnostic groups. Plasma concentrations of P-tau217 (A), GFAP (B), Aβ42/Aβ40 (C), T-tau (D), and NfL (E) were compared between non-DS sibling controls, DS-CS, DS-MCI and DS-dementia. Plasma T-tau and NfL data in overlapping samples have been reported previously. P-values were obtained from univariate general linear modeling, adjusted for age and sex. Statistically significant differences between groups after Bonferroni correction are depicted by one (p < 0.05), two (p < 0.01), or three (p < 0.001) asterisks. Abbreviations: Aβ = β - amyloid; CS = cognitively stable; DS = Down syndrome; GFAP = glial fibrillary acidic protein; MCI = mild cognitive impairment; NfL = neurofilament light chain; P-tau = phosphorylated tau; T - tau = total tau. Copied from Janelidze et al. (2022) under the terms of the Creative Commons Attribution license [66].

Regarding t-tau, Janelidze et al. reported also increased concentrations in demented DS-individuals when compared to non-DS siblings and cognitively stable DS-individuals [66] (see Fig. 3). Additionally, it is worth mentioning that Janelidze and colleagues proved that in participants with DS, plasma p-tau217 levels were consistently associated with abnormal tau-PET and Aβ-PET status in models covaried for age. They concluded that plasma p-tau217 is a very accurate blood-based biomarker of both tau and Aβ pathological brain changes in DS. Plasma p-tau217 might therefore help guide screening and enrichment strategies for the inclusion of individuals with DS in future AD clinical trials, especially when it is combined with age as a covariate [66].

#### 3.1.3 Neurofilament light chain

NfL is one of the scaffolding proteins of the neuronal cytoskeleton inside the subcortical axons. It is released after axonal destruction and reflects damage as a result of neurodegeneration [69].

Plasma NfL levels could serve as a potential biomarker for neurodegeneration in the general population, helping to differentiate cognitive decline caused by AD from other conditions that lead to cognitive impairment during the prodromal stages in the general population [70].

Strydom and colleagues (2018) demonstrated that NFL in blood could be an easily accessible and inexpensive diagnostic marker for the early detection of AD in DS [7].

Plasma NFL concentrations (measured with Simoa® technology) increased with age and were significantly higher in all DS groups as opposed to healthy controls. Furthermore, levels of plasma NFL were higher in both the prodromal AD group and the AD dementia group than in the asymptomatic DS group, supporting the optimal diagnostic performance of NFL to differentiate between DS with and without AD dementia. Since NFL levels increased 20 years before prodromal AD diagnosis [71], before the onset of dementia symptoms, hereby reflecting the degree of neurodegeneration before clinical onset, plasma NFL may aid in the diagnosis of both prodromal AD and AD dementia in DS [72,73]. These results were confirmed in a recent study by Ashton et al. (2021), Janelidze and colleagues [66], and Lleó et al. [67]. They found a substantially higher increase in NFL plasma levels in DS individuals with AD. In a recent study by Fortea et al. (2020), a trend of increased levels of both plasma and CSF NFL in DS patients with AD when compared to healthy DS patients and healthy aged-matched controls was reported [74], as well as in the paper by Fagan and colleagues (2022) [27]. The strong correlation between blood and CSF NFL concentrations in DS confirms the clinical relevance of plasma NFL measurements.

### 3.2 Neuroinflammation-related biomarkers

It is hypothesized that DS inherently results in chronic neuroinflammation, as is the case in all neurodegenerative processes. Neuroinflammation plays an important role in the development and progression of AD in the general population [75]. Microglial and astrocytic activation, increased inflammatory gene expression, formation of immune complexes, and cerebral oxidative stress have been reported to occur at different ages in DS [[76], [77], [78], [79], [80], [81], [82], [83]].

Moreover, Aβ causes an upregulation of pro-inflammatory cytokines by activating microglial cells in the brain [84]. As such, the reduction and/or depletion of microglia has been investigated as a therapeutic avenue for DS, AD, and other neurodegenerative diseases [85]. In addition to amyloid- and tau-related pathology, AD in DS is characterized by neuroinflammatory processes due to overexpression of inflammation-associated genes on chromosome 21, among which Coxsackievirus And Adenovirus Receptor (*CXADR*), A disintegrin and metalloproteinase with thrombospondin motifs 1 (*ADAMTS1*), and superoxide dismutase 1 (*SOD1*). CXADR, for example, can induce stress-activated mitogen-activated protein kinase (MAPK) pathways in the heart. This leads to increased production of various pro-inflammatory cytokines, including interferon gamma (IFNγ), interleukin (IL) 12, IL-1β, tumor necrose factor alpha (TNFα), and IL-6 [86].

#### 3.2.1 Triggering receptor expressed on myeloid cells 2

In the brain, triggering receptor expressed on myeloid cells 2 (TREM2) is exclusively expressed by microglia [[87], [88], [89], [90], [91], [92]] and its expression is modulated by neuroinflammation [93].

The soluble fragment of TREM2 (sTREM2) may act as a marker for microglial activation and has been demonstrated to be lower in plasma in AD patients in the general population when compared to subjects with forms of potential reversible cognitive impairment [94].

TREM2 levels are increased in serum and plasma of young adults with DS, as determined by immunohistochemistry and Western blot [95,96], while older adults exhibit lower levels of TREM2 in serum [95]. Such an expression pattern might represent divergent effects on microglial activity along AD in DS [97].

#### 3.2.2 Glial fibrillary acidic protein

GFAP is an astrocytic cytoskeletal protein that serves as a marker of abnormal activation and proliferation of astrocytes due to neuronal damage, i.e. astrogliosis [98]. Astrogliosis was observed around Aβ plaques from the prodromal stages of AD (i.e. MCI stage) [99], and GFAP expression has been reported to correlate with Aβ plaque density in AD brain tissue [[100], [101], [102]].

Higher plasma levels of GFAP are correlated with lower measures of episodic memory and microstructural integrity, as well as with cortical thinning in AD, MCI, and healthy aged controls, as measured with Simoa® [102,103]. For further information regarding blood GFAP levels as a biomarker for AD, we kindly refer to the following review by Kim and colleagues [38].

Janelidze and colleagues also showed increased plasma levels of GFAP in DS individuals with MCI and AD as compared to non-DS siblings and cognitively stable DS individuals [66]. A recent study indicated that starting in their mid-40s, people with DS have increased levels of GFAP in plasma [104]. This points out that the secondary effect of abnormal protein accumulation changes in the innate immune system. These changes in the innate immune system and neuroinflammation are now considered to be an early core feature of both DS and AD that interfaces with and may contribute to the clinical manifestation of cognitive disorders and further cognitive decline [47,[104], [105], [106]]. Finally, in participants with DS, GFAP was consistently associated with abnormal tau-PET and Aβ-PET status in models covaried for age [66].

#### 3.2.3 Lipocalin-2

NGAL, also known as lipocalin-2, is an acute-phase protein induced by injury, infection, or other inflammatory stimuli, recently identified as a new inflammatory constituent of the pathophysiology of AD [107,108].

Blood NGAL levels are significantly higher in AD patients when compared to healthy controls in the general population [109]. For more information regarding neutrophil activation in AD, we kindly refer the reader to the review of Wu and colleagues [109].

Serum NGAL levels were significantly increased in elderly DS subjects compared to elderly healthy people without DS. The correlation between age and DS was increased, but NGAL levels (as measured with ELISA) were not associated with the clinical diagnosis of dementia, nor the progression to dementia [110]. Importantly, the study by Naudé and colleagues (2015) showed different associations of serum NGAL levels with Aβ_1-40_, Aβ_1-42_, their truncated species, and their ratios. Increased serum NGAL was positively associated with plasma Aβ_1-40_ and Aβ_n-40_ in the non-demented DS group, indicative of the fact that in non-demented DS subjects, Aβ_1-40_ has not yet accumulated into plaques. Additionally, in the demented DS group, increased serum NGAL was positively associated with Aβ_1-42_ and Aβ_n-42_. The increased levels of circulating Aβ_1-42_ peptides were most probably a consequence of the pro-inflammatory environment with microglia activation. Microglia cells can clear Aβ_1-42_ from the brain and additionally, they may increase the production of Aβ_1-42_ via APP. Rather than reflecting the clinical symptoms, serum NGAL reflects the neuropathological alterations in Aβ accumulation and circulation in accordance with the conversion to and progression of dementia in DS. Further investigation is needed to establish if NGAL can improve the sensitivity of plasma Aβ as a biomarker for AD in DS [107]. In non-DS AD individuals, serum and CSF NGAL levels (as measured with a sandwich ELISA) can be used as a biomarker for the conversion of MCI to AD. Whether this is also the case in DS individuals with AD, has yet to be investigated [108].

#### 3.2.4 Pro neurotrophin nerve growth factor

ProNGF is the precursor of neurotrophin nerve growth factor (NGF), a neurotrophin that is amongst other things, crucial for the development and survival of the basal forebrain cholinergic neurons [111]. Both in AD and non-demented DS, deregulation of the NGF metabolic pathway with proNGF accumulation leads to progressive degeneration and dysfunction of these cholinergic neurons [112]. Studies suggested that the NGF metabolism is further compromised by early neuroinflammation and the activation of matrix metalloprotease 9 (MMP-9), which is an NGF-degrading protease [111].

Recent research indicates that proNGF is associated with the pathology of AD; however, its specific use as a blood-based biomarker for this condition has not yet been established in the existing literature.

Iulita and colleagues (2016) investigated whether proNGF in body fluids can be used as a biomarker of cognitive decline in DS. They discovered significantly higher levels of plasma proNGF in DS individuals with overt dementia, but also in asymptomatic AD individuals within the general non-DS population. The elevated levels of proNGF (measured with Western blot) in the AD-asymptomatic stages, support the evidence that other phenomena, besides Aβ deposition, occur earlier in life than anticipated and develop silently over decades. There was no significant correlation between plasma proNGF at baseline and the rate of cognitive decline. However, DS subjects showing increased proNGF levels at follow-up, suffered greater cognitive decline the next year after sampling. In conclusion, combining an inflammatory marker, such as proNGF, with Aβ biomarkers has the potential to be a robust predictor of cognitive decline in DS [113]. Pentz and colleagues (2021) also showed increased proNGF levels in plasma and CSF from individuals with both DS and AD in DS when compared to healthy individuals [114].

#### 3.2.5 Inflammatory cytokines

It has been postulated that local upregulation of pro-inflammatory cytokines and chemokines in the brain is directly involved in the origin of dementia in DS [115,116].

Anti-inflammatory cytokines are significantly increased in plasma of AD patients in the general population [117].

Research findings indicated a tendency towards higher serum and plasma inflammatory proteins in DS subjects compared to healthy controls [118]. DS individuals with AD showed significant changes in plasma levels of TNF-α, IFN-γ, IL-6, IL-8, IL-10, MMP-1 (Matrix Metalloproteinase 1), MMP-3, MMP-9 and MIP-1α (Macrophage inflammatory protein-1 alpha). The levels of matrix metalloproteinases, TNF-α, IL-6, and IL-10 were higher in DS subjects (measured with MSD), even in those without AD. The changes in TNF-α and IL-8 were the strongest predictors of cognitive decline. Plasma levels of IL-8 were higher in DS + AD compared with AD-asymptomatic DS. MMP-3, which Pentz and colleagues have also recently shown to degrade mature NGF, but not its pro-form, was increased in the plasma from people with both DS and AD in DS [114]. When combining pro-inflammatory cytokines, such as TNF-α, with Aβ_1-40_ and Aβ_1-42_ into a combined biomarker complex instead of looking at these markers individually, a stronger association with cognitive abilities has been obtained. There is considerable evidence for the existence of early AD-related CNS inflammation in DS, but further longitudinal studies are desired to confirm the potential role of pro-inflammatory cytokines and chemokines as early biomarkers [113,119].

### 3.3 Oxidative stress markers

AD pathogenesis is complex with multiple, interacting pathophysiological processes, including a disturbed oxidative metabolism [120]. Due to an extra copy of *SOD1*, encoded on chromosome 21, people with DS produce an increasing amount of reactive oxygen species (ROS) in all organs. A link exists between ROS, mitochondria, and faster aging [121]. An exacerbated oxidative stress response with oxidative damage to various molecules has been demonstrated in people with DS, even before the onset of AD. Furthermore, the presence of systemic and excessive oxidative stress may lead to Aβ overproduction in the brain and contribute to the development of AD in DS [120,122]. In the next paragraphs, neopterin, superoxide dismutase, and glutathione peroxidase as biomarkers for oxidative stress in AD in DS will be discussed.

#### 3.3.1 Neopterin

Neopterin is an unconjugated pteridine produced by monocyte-derived macrophages and dendritic cells, which can serve as a marker of systemic inflammation and the cell-mediated immune response [123].

##### 3.3.1.1 Neopterin in blood

Neopterin levels were significantly higher in AD patients compared to healthy controls [124].

According to Coppus and colleagues (2010), people with DS had increased plasma/serum neopterin concentrations due to their chronic state of impaired cellular immunity (as measured with High-Performance Liquid Chromatography (HPLC) and fluorimetric measurements). Demented DS subjects had significantly higher neopterin levels compared to the non-demented DS subjects. The risk of developing dementia was almost doubled in DS individuals with plasma neopterin levels above the median. Therefore, increased neopterin levels could be considered an independent risk factor for dementia [125,126]. Mehta and colleagues demonstrated the lack of correlation between neopterin and Aβ levels, confirming that neopterin levels do not reflect AD neuropathology, but rather activated cellular immunity in the DS population [127,128].

##### 3.3.1.2 Neopterin in urine

Neopterin/creatinine urine levels (measured with HPLC) correlated with changes in cognitive functioning over time, even before AD diagnosis. Results suggest that urine neopterin might be a potential early marker of cognitive decline in DS, and as such may aid in tracking the progression from MCI to AD [122].

#### 3.3.2 Superoxide dismutase and glutathione peroxidase

The complex interaction between the antioxidant system and the cognitive phenotype of DS is not fully understood. Several studies investigated antioxidant enzymes and their association with memory functions, but the results were often unexpected and inconsistent [[129], [130], [131], [132]]. Nonetheless, a relevant relationship between altered free radical metabolism and increased lipid peroxidation and memory deficits is evident. Superoxide dismutase (SOD) (measured by spectrophotometry) and glutathione peroxidase (GPx) (levels determined using the method of Paglia and Valentine based on the NADPH coupled reaction [133]) are antioxidant enzymes, clearing ROS produced in cells.

##### 3.3.2.1 Superoxide dismutase and glutathione peroxidase in blood

People with DS may present with high levels of SOD1, as a result of an extra copy of the *SOD1* gene located on chromosome 21. Contrary to this hypothesis, several studies found SOD1 levels or activity to be normal or even reduced in DS [[129], [130], [131], [132]]. Zis and colleagues (2012) confirmed that *SOD1* expression varies considerably in the DS population. In addition, they demonstrated the potential role of SOD1 as a peripheral biomarker for cognitive performance since SOD1 function is inversely correlated with memory decline over time [132]. It has been suggested that GPx levels are better predictors of cognitive decline since a positive correlation between GPx activity and cognitive performance was described [129]. In this regard, Strydom and colleagues showed that high GPx levels were associated with poorer cognitive outcomes and that SOD1/GPx ratios positively correlated with memory dysfunction [130].

##### 3.3.2.2 Malondialdehyde and 8-hydroxy-29-deoxyguanosine in urine

The urine levels of 8-hydroxy-29-deoxyguanosine (as measured with the 8-OHdG assay [134]), a biomarker of oxidative DNA damage, and malondialdehyde, a marker of lipid peroxidation, were significantly elevated in the urine of children with DS. This high rate of DNA damage reflects increased degenerative processes corresponding with an earlier onset of age-related processes [135]. However, until now there are no studies investigating a possible correlation between MDA or 8OH2dG and cognitive functioning in DS adults. Urinary 8OH2dG levels were increased in the general AD population when compared to healthy controls [136].

##### 3.3.2.3 Isoprostane 8,12-iso-iPF_2_alpha in urine

Isoprostane 8,12-iso-iPF2alpha is a sensitive and specific marker of *in vivo* lipid peroxidation and has been shown to be increased in the general AD population. In the longitudinal study of Zis and colleagues (2014), DS subjects showed elevated levels of urinary iPF2alpha compared to controls. Urine levels were measured using gas chromatography and mass spectrometry. Changes in urinary iPF2alpha over time were associated with more pronounced memory decline. These results suggest that sequential measurements of urinary iPF2alpha have considerable potential as a biomarker for memory decline and progression of MCI to AD in DS [132]. Other studies also revealed the increased *in vivo* lipid peroxidation and pro-oxidant condition early in the course of DS. Pratico and colleagues (2000) reported increased (amounts of) urinary 8,12-iso-iPF2 α-VI levels, that positively correlated with age and disease duration [120]. Manna and colleagues (2016) demonstrated a significant increase in plasma F2-isoprostanes, F2-dihomo-isoprostanes, and F4-isoprostanes, respectively markers of systemic, white and grey brain matter lipid peroxidation, inversely correlating with memory functioning [137]. However, not all studies support the theory of oxidative stress and lipid peroxidation. For instance, Tolun and colleagues (2012) found no significant differences regarding urinary iPF2alpha or allantoin levels between DS subjects and control individuals. They concluded that systemic oxidative stress may not necessarily be present in DS, nor can it always be reliably measured [138].

### 3.4 Monoaminergic and amino acid markers

#### 3.4.1 Monoamines

The monoaminergic system encompasses the catecholamines (dopamine, noradrenaline, and adrenaline) and the indolamines (e.g. serotonin). Various studies have described a degeneration of monoaminergic cells and substantial changes in the levels of monoamines and their metabolites in AD and DS brains [[139], [140], [141], [142]].

There are publications indicating altered levels of monoamines in the plasma of AD patients compared to the general population [143,144].

Dekker and colleagues (2015, 2018) analyzed biogenic amines and their metabolites in the serum of DS individuals without AD, with prodromal AD (who converted over time), and with clinically diagnosed AD. The most remarkable results were the significantly lower serum levels of 3-methoxy-4-hydroxyphenylglycol (MHPG), the primary noradrenergic metabolite, in demented subjects with DS and in subjects with DS who later converted to dementia compared to non-demented DS subjects and healthy controls. Serum MHPG was therefore proposed as a potential biomarker for the development of AD in DS. Non-demented DS individuals had a 10-fold increased risk of developing dementia when their MHPG levels were below the median. In addition to the group differences in MHPG levels, various other monoaminergic compounds varied between the three diagnostic groups [144,145]. In summary, there is an ongoing process of monoamine alterations in DS associated with the absence or presence of, or progression to AD. The monoamine composition already differed significantly years before the clinical diagnosis of dementia (in the prodromal stage) [144]. However, a previous study, performed by Coppus and colleagues, found a significant increase in plasma homovanillic acid (HVA, dopamine metabolite) in DS subjects compared to controls, indicating that the dopaminergic system is affected in DS, at least in the non-demented population [123]. A follow-up study is necessary to unravel the cause for the discrepancies found between plasma and serum and to investigate the clinical utility of monoamine neurotransmitters as predictive markers of AD in DS.

#### 3.4.2 Amino acids

It has been shown that AD patients often exhibit altered plasma concentrations of various amino acids. Some amino acids may be significantly reduced, while others may be elevated compared to healthy controls [146].

Metabolomics has recently emerged as a new research platform to provide new insights into AD pathogenesis and to discover diagnostic AD biomarkers [147]. Coppus and colleagues investigated the spectrum of amino acids as neurotransmitters in DS subjects related to their clinical dementia status. Plasma levels of nearly all amino acids differed substantially from healthy controls (general population). Methionine, glutamate, and all large neutral amino acids (LNAA) were significantly decreased in DS individuals, while taurine and glycine were significantly increased compared to controls. A few amino acids appeared to have the potential to differentiate between demented DS subjects and non-demented subjects. In the demented group, an additional increase of taurine was observed, but this was also described in AD, PD, and other neurodegenerative diseases. Moreover, the plasma concentration of phenylalanine was significantly higher in demented DS individuals compared with healthy subjects [123,126].

### 3.5 Epigenetic markers

Epigenetic mechanisms, such as DNA methylation and posttranslational histone modifications, play a crucial role in gene expression without altering the DNA sequence itself. The extra copy of chromosome 21 in DS can disrupt the normal epigenetic processes due to changes in gene dosage. It is hypothesized that these epigenetic alterations contribute to premature aging and can be linked to the development and severity of cognitive impairment in DS [148,149].

A recent study by Obeid and colleagues (2016) showed that plasma concentrations of SAM, SAH, and choline were significantly higher in DS subjects, whereas the SAM/SAH ratio was reduced compared to controls. The higher concentration of SAH was associated with hypomethylation of ASPA and ITGA2B. These two CpG sites became hypomethylated with increasing age [150]. Jones and colleagues (2013) were the first to investigate the association between altered DNA methylation patterns and cognitive functioning. They examined DNA in buccal epithelial cells of DS individuals and healthy controls. They found 3300 CpG sites differently methylated for more than 10 % between DS and controls. Five of these CpG sites positively correlated with cognitive function, indicating that DS subjects with cognitive impairment have additional DNA methylation disruptions. DS is characterized by a tendency of hypermethylation since more than 66 % of the altered CpG sites were hypermethylated compared to controls. In longitudinal studies on patients with DS, the evolution of DNA methylation changes related to cognitive functioning is studied, and whether these changes can be used as predictive markers of cognitive decline is defined [151]. It is however essential to mention that measuring epigenetic markers in blood is a more complex procedure than used for the other biomarkers for AD in DS.

## 4 Potential future candidate peripheral fluid biomarkers

In the following paragraphs, we will focus on biomarkers relevant for AD in the general population, that could also become applicable to AD in DS. For this reason, we took inspiration from candidate biofluid biomarkers recently described in the AD population.

### 4.1 Fibrinogen β chain, alpha-2-HS-glycoprotein G, fibrinogen α chain and plasma protease C1 inhibitor

A recent study performed by Kobe and colleagues (2020), investigated the presence of four peptides in the serum of healthy age-matched controls, patients with MCI, and patients with AD. They detected peptides of fibrinogen β chain (FBC), alpha-2-HS-glycoprotein (AHSG), fibrinogen α chain (FAC), and plasma protease C1 Inhibitor (PPC1I). FBC and FAC are essential coagulation factors and key contributors to AD pathology. PPC1I regulates coagulation and neuroinflammation in a damaged brain, while AHSG is anti-inflammatory, and regulated under the control of TNFα. Next, they analyzed the presence of the mother proteins in brain tissue (with immunohistochemistry) and found an upregulation of FBC, FAC, and PPC1I, and a downregulation of AHSG [152]. Since DS is accompanied by neuroinflammation, these proteins could also reflect the level of neuroinflammation in AD in DS.

### 4.2 Intercellular adhesion molecule 1 and vascular cell adhesion Molecule-1

Intercellular adhesion molecule 1 (ICAM-1) and vascular cell adhesion molecule-1 (VCAM-1) are cell-surface glycoproteins on endothelial cells and immune cells, mediating the adhesion of leukocytes to endothelial cells and the transport of leukocytes to the brain. Zuliani and colleagues (2008) reported increased plasma levels of VCAM-1 (as measured by ELISA) compared to non-demented control patients [153]. Rentzos and colleagues (2004) found increased levels of serum-soluble ICAM-1 compared to non-inflammatory neurological diseases and non-diseased control individuals [154,155]. Given the development of neuroinflammation in DS, ICAM-1 and VCAM-1 could thus be potential biomarkers for AD in DS.

### 4.3 Interleukine-33

Interleukine 33 (IL-33) and the soluble form of its receptor ST2 (sST2) have been linked to inflammation in AD. IL-33 levels are elevated in plasma when comparing MCI and AD to healthy controls [156,157]. Higher levels of IL-33 in serum were linked to better performance on cognitive tests in a one-year follow-up, as measured by Western Blot [157]. The fact that these higher levels are associated with better cognitive functioning is counter-intuitive since patients with AD and MCI have higher levels than healthy controls. Saresella and colleagues (2020) and Liang and colleagues (2020) showed that AD patients have elevated levels of sST2 (measured by ELISA), which ameliorates the physiological effects of IL-33 and can contribute to the decline of cognitive function in the course of AD [156,157].

### 4.4 Neurogranin

Neurogranin (Ng) is a post-synaptic protein involved in memory formation. A link between AD and Ng is thus not far-fetched. CSF Ng levels are elevated in AD when compared to healthy controls and these levels correlate with cognition [[158], [159], [160]]. Contrarily, the Ng levels in blood plasma exosomes were decreased in individuals with AD and MCI when compared to healthy controls [160].

### 4.5 Dual-specificity tyrosine-regulated kinase 1A

Dual-specificity tyrosine-regulated kinase 1A *(DYRK1A)* is an important gene involved in the etiology of both AD and DS [161]. It is mapped to the so-called Down syndrome critical region, a restricted region on chromosome 21 that is considered necessary and sufficient to cause the DS phenotype. As a result of trisomy 21, there is a 1.5-fold increase in DYRK1A mRNA and protein levels [162]. It interacts with many neurodegeneration-linked proteins such as APP, MAPT, PSEN1, and (Alpha Synuclein) SNCA [163]. In the paragraphs below, we will discuss these involvements more closely.

*DYRK1A* expression is abnormally high in sporadic AD when compared to healthy controls (as detected in brain tissue with Western Blot) [164]. Its levels are decreased in plasma when comparing healthy controls to AD patients [165,166].

As previously mentioned, DYRK1A interacts with the APP. On this note, García-Cerro and colleagues (2017) proved that genetic correction of *DYRK1A* in a *Dyrk1a* mouse model normalized APP and Aβ levels [167]. DYRK1A was shown to phosphorylate APP and enhance its cleavage by β- and γ-secretases *in vitro* and mammalian cells. This enhances the formation of Aβ_1-40_ and Aβ_1-42_ and eventually amyloid plaques [168]. It is also worth mentioning that Fernandez Bessone and colleagues (2022) highlighted DYRK1A as a regulator of APP axonal transport and metabolism. With live cell imaging in human-derived neurons, they found that DYRK1A activity differentially regulates the intracellular trafficking of the APP. Single particle analysis revealed DYRK1A as a modulator of axonal transport and the configuration of active motors within the APP vesicles [169].

DYRK1A also causes phosphorylation of tau; At 11 locations to be precise, including Thr181, Ser199, Ser202, Thr205, Thr212, Thr217, Thr231, Ser396, Ser400, Ser404 and Ser422 [170]. These sites are phosphorylated in adult DS brains, but not in age-matched controls [171]. Abnormal phosphorylation causes the loss of axonal transport and promotes tau self-aggregation and fibrillization. Furthermore, DYRK1A enhances tau expression through stabilizing mRNA coding for tau [168]. Neuropathological and molecular studies indicate that overexpressed nuclear DYRK1A contributes to the modification of the alternative splicing of tau and neurofibrillary degeneration. DYRK1A phosphorylates the alternative splicing factor (ASF) [172]. DYRK1A phosphorylation of the ASF drives the splice factor into nuclear speckles while it also prevents ASF-mediated inclusion of the alternatively spliced exon 10 in tau mRNA [173]. Moreover, phosphorylation of ASF by DYRK1A inhibits their association with nascent tau transcripts, thus increasing 3R-tau levels and causing an imbalance of the 3R-4R tau isoforms [168].

### 4.6 Telomere length

Telomeres are arrays of highly conserved ‘TTAGGG’ repeats at the terminal ends of chromosomes, that become shorter with each cell division [174]. Cognitive and functional decline due to AD has been linked to accelerated shortening of telomeres in the general AD population [175]. Jenkins and colleagues (2008, 2010, 2012, 2016, 2017) investigated telomere length in T-lymphocytes from individuals with DS with and without dementia, to discover whether telomere length could serve as a valid biomarker for clinical progression to AD. Consistent telomere shortening over time was observed in all diagnostic groups (cases and controls). Differences in telomere length between DS and healthy controls emerged before the presentation of clinical dementia signs in DS. At the moment of MCI or AD diagnosis, individuals with DS had shorter telomere lengths than the age- and gender-matched control group. DS subjects with MCI or AD both had shorter telomeres compared to unaffected DS individuals. These results demonstrate a significant association between telomere shortening and declining clinical status reflective of AD progression. The longitudinal and cross-sectional findings of these studies suggest that telomere length might be an informative biomarker to accurately monitor the transition from clinically normal to dementia [[176], [177], [178], [179], [180]]. We do want to stress that the complexity of the technical and analytical aspects is much greater for telomere length as a biomarker than other potential peripheral biomarkers for AD in DS.

**Fig. 4:**
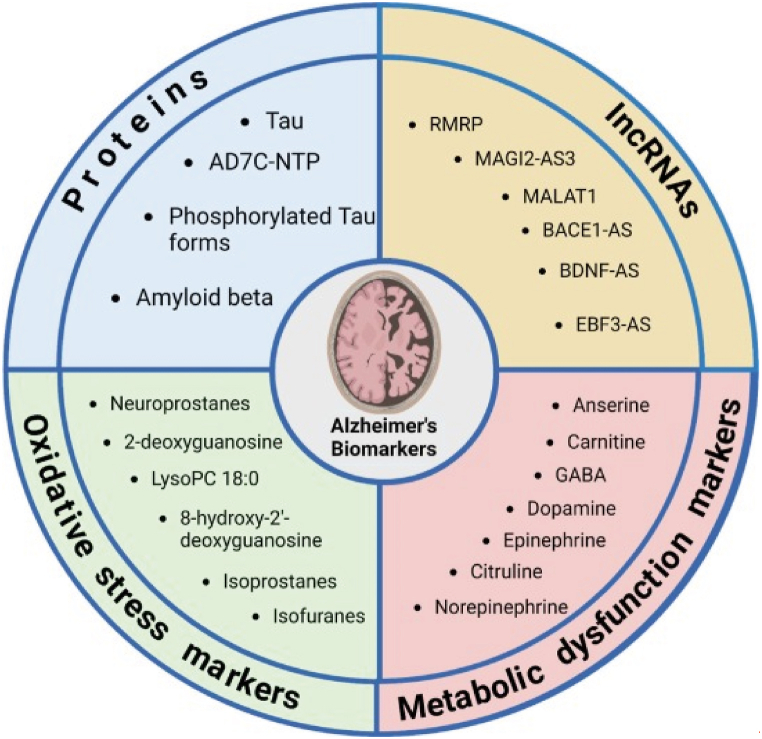
AD-related biomarkers in blood and urine samples that could be integrated into WBE surveillance systems. They are organized in four main categories: proteins, lncRNAs, oxidative stress markers, and metabolism dysfunction markers. Reused from Ref. [136] according to the Attribution-NonCommercial-NoDerivatives 4.0 International CC BY-NC-ND 4.0 Deed. https://creativecommons.org/licenses/by-nc-nd/4.0/.

Fig. 4 (Armenta-Castro et al. [136]) summarizes the most promising urine biomarkers for AD in general. The ones that we discussed before in blood throughout this review, could also be used as potential biomarkers for AD in DS.

Aβ and Tau, which are probably the most studied biomarkers in AD and also in AD in DS, can be found in the urine of both AD patients and control individuals [181]. Therefore it would be relevant to determine whether these potential biomarkers could also differentiate between DS patients with(out) AD, using techniques like ELISA and Western Blot [182,183]. Also several Neural thread proteins (NTPs) are useful to distinguish between AD and non-AD patients. AD7c-NTP is the most commonly studied one as it is associated with pTau immunoreactive cytoskeletal lesions and neurite sprouting [[184], [185], [186]]. Another interesting protein we would like to mention is Nerve Growth Factor Receptor (NGFR), more specifically, the levels of truncated forms of NGFR which are increased in blood and urine of AD-patients and which are linked to neuronal loss [187]. A second category of potentially interesting urine-based biomarkers are the long non-coding RNAs (lncRNAs). Multiple lncRNAs are linked to genes involved in neuroinflammation, apoptosis and APP cleavage. A summary of the most interesting lncRNAs can be found in Fig. 4. We would also like to kindly refer to table 4 of the review by Armenta-Castro et al. [136] which summarizes these lncRNAs with reference to their linked gene. The third category encompasses oxidative stress markers, including isoprostanes and isofuranes, and oxidized nucleic acids and proteins, and a final category encludes metabolic dysfunction markers, among which several catecholamines and their metabolites, as well as amino acids [136].

## 5 Conclusion and future directions

**Fig. 5:**
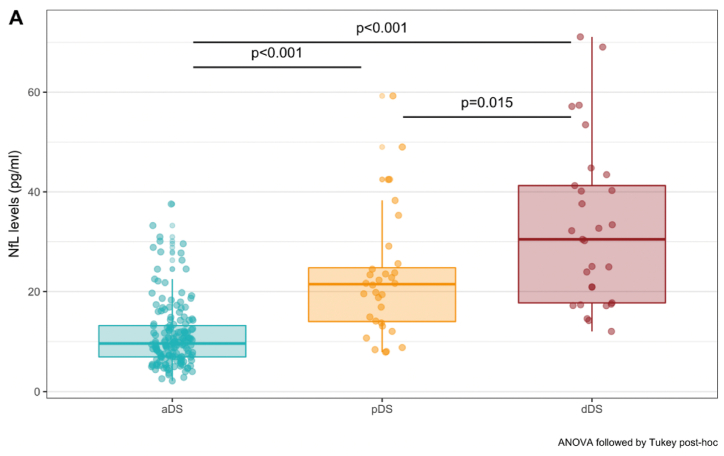
Baseline plasma NfL levels across baseline diagnostic categories. Age, sex, and intellectual disability were included as covariates in the analysis. Only statistically significant associations are shown. aDS: asymptomatic Down syndrome (n = 165); pDS: prodromal Alzheimer's disease in Down syndrome (n = 32); dDS: Alzheimer's disease dementia in Down syndrome (n = 29). Image used with permission for reuse from Ref. [47].

There are multiple candidate biomarkers available that need further investigation before implementation in daily clinical practice. Additionally, there is a need for biomarkers (next to Aβ_1-40_ and Aβ_1-42_) that reflect Aβ deposits in blood vessels which could cause CAA and microbleeds or hemorraghia. AD in DS is also associated with CNS inflammation in its early stages, and it appears that neuroinflammatory markers can improve the diagnostic sensitivity of plasma Aβ as a biomarker. As a result, combining inflammatory markers with amyloid biomarkers could hypothetically induce stronger indications for cognitive dysfunction. Research suggests that oxidative stress increases in DS during aging and precedes the onset of clinical AD. Oxidative stress markers in blood and urine show remarkable potential to identify prodromal and dementia stages of AD in people with DS. One of the most promising peripheral markers for early detection of AD in DS seems to be plasma NfL. Fig. 5 shows the differences in NfL levels between asymptomatic DS, prodromal AD in DS and AD in DS.

Additionally, there is significant proof for alterations in serum levels of monoamine neurotransmitters and amino acids in DS which might be related to dementia status. Often one of the largest restrictions of these studies is the limited sample size. The ethical stigma of research on DS individuals hampers the recruitment of participants, especially in obtaining sufficient biosamples. Rather than overprotecting them, we need to give DS individuals and their caregivers the possibility to participate in clinical research. By raising awareness, this might be possible. By combining clinical care and scientific research, more samples can be actively recruited to enlarge research possibilities. For instance, blood samples for biomarker studies can be obtained along with the samples for routine laboratory tests. Several potential biomarkers have been studied in DS and by organizing larger multicenter longitudinal studies in the future more markers will be identified. To be able to predict and diagnose dementia in DS in clinical practice, biomarker levels in correlation with cognitive status need to be confirmed in large DS cohorts with longitudinal follow-up and cut-off values for sensitivity and specificity need to be established. And finally, biomarker discovery can of course only progress with concurrent technology development or further improvements to existing technologies.

## CRediT authorship contribution statement

**Charlotte Jacob:** Writing – original draft, Investigation, Conceptualization. **Marleen Tollenaere:** Resources, Project administration. **Hanane Kachar:** Writing – original draft. **Marie-Claude Potier:** Writing – review & editing. **Peter Paul De Deyn:** Writing – review & editing, Funding acquisition, Conceptualization. **Debby Van Dam:** Writing – review & editing, Supervision, Funding acquisition, Conceptualization.

## Ethics approval and consent to participate

Not applicable.

## Consent for publication

No person's data was involved in this paper.

## Data availability

Not applicable.

## Declaration of competing interest

Authors declare that they have no known competing financial interests or personal relationships that could have appeared to influence the work reported in this paper.
